# The double-edged effect of bank liquidity creation efficiency on systemic risk: Evidence from China

**DOI:** 10.1371/journal.pone.0313208

**Published:** 2024-11-14

**Authors:** Shuying Tan, Tingting Liu, Chan Wang

**Affiliations:** 1 School of Economics and Finance, Chongqing University of Technology, Chongqing, China; 2 School of Finance, Shanghai University of Finance and Economics, Shanghai, China; Abu Dhabi University, UNITED ARAB EMIRATES

## Abstract

Bank liquidity creation fosters economic growth while entailing liquidity mismatch risk. This study explores whether improving bank efficiency during liquidity creation can mitigate systemic risks. We propose a bank liquidity creation efficiency measure based on a stochastic frontier approach to analyze Chinese commercial listed banks from 2000-2021. Through systemic risk decomposition, we find that higher profit efficiency alleviates individual tail risk but strengthens system linkage for Chinese banks, representing a double-edged effect. Further analysis reveals that high profit efficiency is associated with more investment in real estate and higher risk-taking on loans. Banks’ digital transformation can help alleviate the dilemma of being too connected to the system. The results provide implications for emerging markets to prevent risk and promote economic growth.

## 1 Introduction

The bank run experienced by Silicon Valley Bank on March 10, 2023, undermines the stability of global financial markets, thereby highlighting the risk of term mismatch during the bank liquidity creation process. According to the modern theory of financial intermediation, banks exist because they perform two central roles in the economy—they create liquidity and transform risk, which are both crucial to economic growth. Promoting economic development and preventing risks are two-pronged goals for most countries’ markets worldwide, particularly in the aftermath of the COVID-19 pandemic. As a vital part of the financial system, banks create liquidity to foster economic development, as posited by modern financial theory [1, 2]. However, the liquidity creation process is inevitably accompanied by a transformation of risk [3], resulting in a potential trade-off between the two-pronged objectives for banks during that process.

Bank efficiency may influence financial risk and economic growth [4–6]. Hence, improving efficiency during the liquidity creation process of banks may be a key to unifying the dual purpose. However, surprisingly, since Berger and Bouwman [7]proposed a widely accepted measure of bank liquidity creation that describes bank activities comprehensively, little is known about how bank liquidity creation efficiency affects systemic risk or influences modes through which banking services impact the real economy. This paper tries to fill this gap by providing evidence in China, where the financial market is heavily bank-based [8].

Stochastic Frontier Analysis (SFA), by decomposing the error term into a stochastic error to reflect statistical noise and a one-sided error to reflect inefficiency level, can effectively overcome the limitation of treating various measurement errors as inefficiencies. It is a widely adopted method in efficiency evaluation [4, 6, 9]. We employ SFA to measure the liquidity creation cost efficiency and profit efficiency, with bank output defined as the level of liquidity creation, as it comprehensively reflects the operational conditions of banks. We utilize the market-based systemic risk measure developed by Van Oordt and Zhou [10] to gauge systemic risk. The essential advantage of the market-based approach is that it enables us to decompose the systemic risk of individual banks into bank-specific tail risk and systemic linkage to severe shocks in the financial system.

Using data from China’s commercial listed banks over 2000–2021 collected from the China Stock Market and China Stock Market & Database (CSMAR) and the Wind database, we reach results that improving cost efficiency in liquidity creation does not significantly impact systemic risk. However, enhancing profit efficiency in liquidity creation presents a double-edged sword effect on risk. While it reduces individual risk, it concurrently increases linkage to the system, constituting a potential risk accumulation. This stems from how banks augment profits, elevating their risk-taking level and increasing credit exposure to the real estate sector. Luckily, the degree of digital transformation in banks can effectively mitigate systemic connectivity.

This paper aims to theoretically fill a gap in the literature in the following aspects. First, we select systemic risk indicators to comprehensively analyze the complete picture of bank liquidity creation efficiency’s risk characteristics. Previous literature has primarily focused on the relationship between bank efficiency and micro-level banking risks. Moreover, the conclusions lack comparability due to the diversity in the proxy for banking risks. We approach the issue from the perspective of systemic risk by decomposing it into individual tail risk and systemic linkage. It allows for the simultaneous consideration of both macro and micro risks. Second, our empirical results enriched the research conclusions on bank efficiency and risk by dissecting systemic risk. Previous research points out that bank efficiency and risk may correlate through various channels, and there is no congruent evidence regarding the direction of that correlation [5, 6, 11, 12]. We underscore the dual-edged sword effect of efficiency improvement in reducing overall systemic risk and find the dark side of bank liquidity creation profit efficiency. We decompose systemic risk into two complementary components according to van Oordt and Zhou [10] and find that higher bank liquidity creation profit efficiency mitigates individual risk while amplifying linkage to the system. Third, this paper offers explanations from the Chinese context regarding the channels through which risk is generated and methods for risk mitigation, supplementing related literature. Chinese banks’ approach to enhancing profit efficiency heavily relies on real estate mortgage loans. The deep entanglement between banks and the real estate sector has historically formed a substantial source of bank profits. However, this reliance may also sow the seeds of potential risks. If the real estate industry experiences defaults or sudden collapses, it could significantly elevate banks’ liabilities, triggering a contagion of market risks. Then, we corroborated the significant value of digital transformation in the banking sector by assessing the impact of digital transformation on the trade-off between bank systemic risk and liquidity creation efficiency. Digital transformation can mitigate the adverse effects of enhanced systemic connectivity resulting from banks’ efforts to improve profit efficiency.

The conclusions carry rich policy implications empirically. From the perspective of regulation, our research provided managerial wisdom for formulating banking regulatory policies. For one thing, a bank’s individual tail risk and systemic linkage risk are not entirely independent; improving liquidity efficiency will simultaneously impact both, necessitating the coordination implementation of macro-prudential and micro-prudential supervision. For another, we identified real estate loans as a source of risk by further exploring the dark side of the growth process in bank profit efficiency. This insight offers new perspectives and approaches for constructing comprehensive and effective regulatory mechanisms to prevent financial risks. However, the systematic risk measurement approach based on market data confines the research sample of this paper to listed banks only, limiting the generalizability of the conclusions. The research findings, therefore, may not comprehensively cover the entire operational landscape of China’s banking sector.

The remainder of the paper is organized as follows. In Section 2, we review the existing literature and develop the hypotheses. Section 3 describes the data and model specification details. In addition, we focus on measures of the bank liquidity creation efficiency and bank systemic risk decomposition. Sections 4 and 5 present the main results with robustness checks. Section 6 does an extension analysis of the role of digital transformation on efficiency and risk. Section 7 concludes our findings and proposes implications for bank operations and risk management.

## 2 Literature review and hypothesis development

### 2.1 Literature review

Long-term focus on systemic risks and efficiency within the commercial banking sector has garnered extensive academic attention. Despite the established interest in the relationship between bank efficiency and risk, existing literature remains theoretically inconsistent and yields mixed findings regarding the impact of efficiency on risk. Variations in findings might arise from differences in variable measurement, analytical perspectives, and sample selection. The literature primarily bifurcates into two streams. One suggests that banking efficiency could mitigate risks. We collectively conclude this first set of theories as the “mitigating role of bank efficiency”.

Concerning the enhancement of efficiency to reduce risk, Dima et al. [11] identified a positive effect of bank efficiency on bank stability. Drawing on a sample of European commercial banks, Fiordelisi et al. [5]employed Granger causality tests to reveal that inefficient banks often exhibit higher risk levels, emphasizing that improving bank efficiency contributes to increased capital and enhances financial system stability. Saeed et al. [12]found that higher cost efficiency implies lower conventional banking risks. The improvement of bank efficiency also aids in reducing non-performing loan ratios [13]. Profitable banks have more diversified products and loan options to reduce risk [14]. Research around the crisis has also discovered the positive effects of efficiency in surviving crises. Based on a sample of U.S. banks, Assaf et al. [4] found that enhancing cost efficiency during normal periods has a proactive effect, effectively improving a bank’s profitability during crises and reducing the probability of bank default. Moreover, Beccalli et al. [15] and Mirzaei et al. [16] found that banks with high operational efficiency exhibit better stock performance in both normal and crisis periods.

Another strand of literature points out that higher efficiency is associated with higher risk. We collectively conclude this second set of theories as the “efficiency as potential hints of risk”. Higher efficiency levels could have been achieved at the cost of higher risk levels [16]. Bank’s appetite for taking higher risks increases [17]. Specifically, the process of enhancing efficiency may entail the aggregation of risk factors. Do Van Anh [18] observed that high-efficiency banks typically maintain lower capital levels. Moreover, a bank’s inefficiency often indicates lower asset quality, thereby increasing the likelihood of risk [19, 20]. The long-term relationship between a bank’s efficiency and non-performing loan ratios involves a delicate balance, contributing to an elevated risk of bank default [21]. Li et al. [22]even posit that bank efficiency can serve as an early warning indicator for the probability of a bank’s future bankruptcy. Beyond individual bank bankruptcy risk, Isik and Uygur [23] found that the level of efficiency in banks also acts as a forward-looking indicator of macroeconomic crises.

The existing literature has laid a solid foundation for research in this field but provides contradictory predictions. We posit that there remains ample room for further expansion and deepening of the investigation into this topic. Firstly, existing research on banking efficiency mostly tends to exhibit a single-dimension efficiency analysis, focusing either on cost efficiency or profit efficiency. Furthermore, there is a lack of uniformity in output settings while estimating efficiency, which results in conclusions that are deficient in comparability. Secondly, when analyzing the relationship between banking efficiency and risk, there is no consensus on the choice of the risk proxy. Most research focuses on the micro-level risks of individual banking entities, such as non-performing loan ratio, banks’ likelihood of default obtained from Moodys’KMV, and insolvency risk, and lacks attention to macro-level risks. Our paper makes several contributions to the literature on the relationship between bank efficiency and risk. First, unlike previous studies, we estimate both cost and profit efficiency estimators. We do this because cost and revenue efficiencies reflect two different managerial abilities. We posit that each of these efficiencies can have a distinct link with bank systemic risk. Moreover, this paper delves into research inconsistencies by setting output as liquidity creation when measuring efficiency. Liquidity creation serves as a comprehensive metric for measuring bank output and offers an integrated depiction of the overall operational situation of banks, which facilitates a more comprehensive and integrated comparison of bank efficiency, eliminating biases in conclusions that may arise from the selection of measurement scopes.

Second, this paper provides deeper insight into the relationship between bank liquidity creation efficiency and risk based on an anatomy of systemic risk. Previous studies have primarily analyzed the risk-bearing aspects of banks in pursuit of efficiency. Risk-bearing corresponds to the individual dimension of bank risk. There is a lack of existing literature explicitly addressing the relationship between efficiency and macro-level risk. The soundness of the banking sector is a vital ingredient of the financial system’s stability. This paper’s measurement of systemic risk breaks down risk into individual risk and systemic connectivity, aiding in a more precise and comprehensive delineation of the sources of risk.

Third, we investigate the underlying transmission mechanism through which risk is generated and prove the effect of digital transformation on balance efficiency enhancement and risk accumulation for banks. Prior studies showed that bank efficiency affects firms’ borrowing costs [8]and productivity [14], thus influencing economic growth [7]. The observed phenomenon in our study points out that enhanced profit efficiency is attributed to more intense risk-taking behaviour by banks, with loan allocations leaning towards the real estate sector. The ongoing digital transformation of commercial banks is conducive to reducing the connectivity between individual banks and the system, thus avoiding the impact of systemic risk.

### 2.2 Hypothesis development

Competition seems to be a significant driving force among the banks’ long-run stability determinants. The competitiveness of a bank is highly related to bank cost efficiency. Competitive banks are more likely to implement better monitoring mechanisms while liquidity creating and hence are less likely to suffer non-performing loans [24]. Also, these competitive banks can extract monopolistic rents from markets as reflected by higher interest rates charged on loans and lower interest rates offered for deposits [25, 26]. The process of transforming liquid deposits into illiquid liabilities is the core of bank liquidity creation, thus performing high liquidity creation profit efficiency. On the whole, efficient banks tend to be more competitive, which enhances bank stability and reduces the likelihood of exposure to systemic risk. As a result, Hypothesis 1 is formulated.

***H1*:** Bank liquidity creation efficiency will reduce the bank’s overall systematic risk.

As early as 1997, Berger and De Young [26] point out that bank efficiency may be an important indicator of future problem loans. Low inefficient banks may tend to have loan performance problems due to poor senior management, which resulted in difficulties in monitoring both their costs and their loan customers, with the losses of capital generated by both these phenomena potentially even leading to failure. Larger banks can control their operating costs through better management and operational procedures and transfer the various categories of risks [11]. Low levels of efficiency could lead banks to try to boost returns by lowering their operating standards, such as less intensive credit monitoring.

***H2(high management level hypothesis)*:** High bank efficiency is an indicator of high management quality.

However, banks can also simply take on higher risks to compensate for lost returns and increase revenue, thus increasing profit efficiency [5]. High-profit efficiency during normal times may reflect excessive risk-taking that earns high returns in normal times but will create problems in subsequent crises [4]. High-risk investments generally have higher returns. To illustrate, banks’ investments in mortgage-backed securities (MBS) were remarkably profitable during the normal period before the subprime financial crisis. Still, they proved very risky and a significant contributing factor to bank failures and the crisis.

***H3(high risk-taking appetize hypothesis)*:** High bank efficiency is a reflection of high risk-taking appetite.

## 3 Methodology, variables and data

### 3.1 Variable estimation

#### 3.1.1 Bank liquidity creation

Liquidity transformation is considered the preeminent function of commercial banks. As the liquidity creator of society, the fundamental task is accepting short-term, liquid deposits and making long-term, illiquid loans, which directly facilitate production by affecting the successful channelling of savings to investment. An intuition for this function is that banks create liquidity by holding illiquid items in place of the non-bank public and giving the public liquid items. Bank liquidity creation measures how banks perform this function by focusing on liquidity classification and weight-assigning balance sheet items.

This study adopts the “Three-step methodology” as introduced by Berger and Bouwman [7] for the comprehensive measures of bank liquidity creation, categorizing balance sheet banking activities into three liquidity classes: liquid, semi-liquid, and illiquid. S1 Table provides the division standards details. A commercial bank’s liquidity creation is quantified through a weighted summation of these classifications, with the formula expressed as follows.

$$\begin{array}{c} \begin{array}{cc} LC= & 1/2*(illiquidassets+liquidliabilities)+0*(semiliquidassets+semiliquidliabilities)\\ & -1/2*(liquidassets+illiquidliabilities+equity) \end{array} \end{array}$$

**Fig 1:**
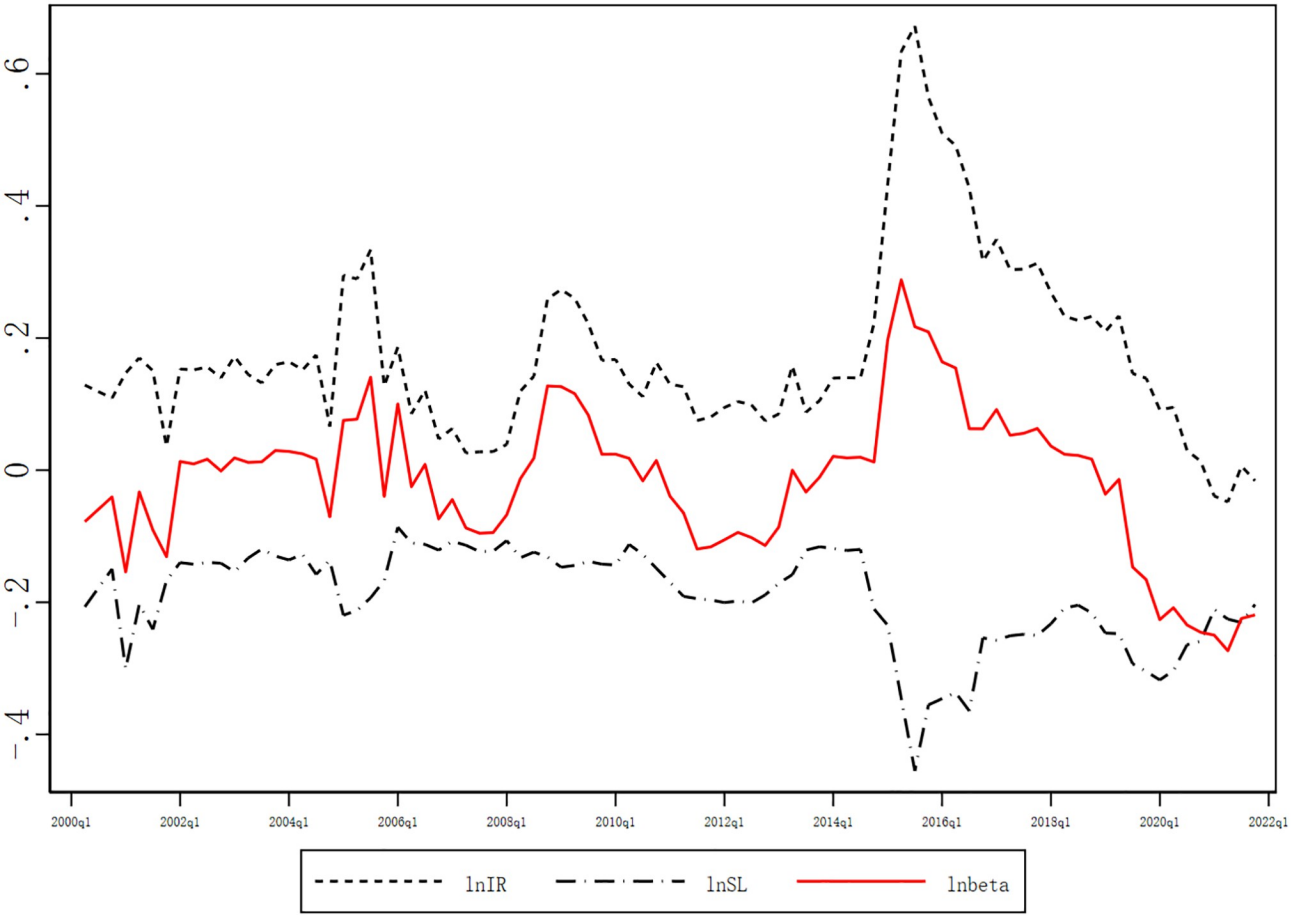
Year trend of LC_TA for different bank ownership structures. This figure plots quarterly estimation results of LC_TA for different bank types within the sample period.

The measure helps us gain a deeper insight into banks’ role as liquidity creators and risk transformers. Considering the operational characteristics of Chinese banks, we draw on existing influential research [27] to adjust liquidity category and empowerment. Fig 1 shows the times series data of the liquidity creation scaled by assets(LC_TA) for different bank ownership structures since 2010. In the initial stages, state-owned and joint-stock banks exhibited a higher level of liquidity creation per unit of asset. However, in recent years, the capacity of rural commercial banks to serve the real economy has rapidly improved, increasingly approximating the levels observed in joint-stock banks. Building upon these shifts, a pivotal area for subsequent analysis will be the cost efficiency and profit efficiency of different types of banks in the liquidity creation process.

#### 3.1.2 Bank liquidity creation efficiency

The concept of efficiency originates from measuring the achievement of bank economic goals by researchers of the last three decades. Banks often pursue the economic goals of cost minimization or profit maximization. The bank liquidity creation cost/profit efficiency tells us how close a bank’s cost/profit is to what a best-practice bank’s cost /profit, which operates in close proximity to the efficient production frontier, for producing the same current level of liquidity creation under the same conditions.

Commercial banks can fundamentally be conceptualized as input-output systems that produce financial products by incorporating essential factors such as fixed assets, human resources, and capital. In calculating bank efficiency, the most critical and preliminary step is the specification of input and output variables within the cost and profit frontier functions. We define bank total profit as pre-tax net income and total costs as the sum of interest expenses, fee and commission expenses, operating expenses, and other operating expenses. While there is a consensus within academic research regarding the inputs of commercial banks, there remains a disparity concerning the definition of outputs. Acknowledging the role of banks in the liquidity creation process, this paper innovatively posits liquidity creation as the bank’s output. Liquidity creation serves as a comprehensive measure of a bank’s overall operations. This setting has two-fold advantages. On the one hand, liquidity creation outperforms loans in reflecting how banks promote real economic growth [7, 28]. On the other hand, the calculation of liquidity creation is relatively standardized worldwide, making the efficiency measure more comparable across countries. We specify three input prices: *w*_1_, the price of purchased funds (the ratio of total interest expenses to total liabilities); *w*_2_, the price of labor (ratio of total personnel expenses to total assets); *w*_3_, the price of physical capital (total operating and administrative expenses to total assets). We also include quantities of two fixed net puts (inputs or outputs): *z*_1_, the total value of total bank off-balance sheet activities and *z*_2_, bank financial equity capital. The details are listed in Table 1.

**Table 1:** Input-output variable definitions of bank efficiency.

Settings	Symbol	Variables	Definitions
Total profit	PI	Profit	Net income before tax
Total cost	TC	Total cost	The sum of interest expenses, fee and commission expenses, operating expenses, and other operating expenses
Output Y	LC	Liquidity creation	Value of liquidity creation
Input price *W*	w1	Price of funds	Total interest expenses/total liabilities
	w2	Price of labor	Personnel expenses/total assets
	w3	Price of physical capital	(Administrative expenses-personnel expenses-fixed assets depreciation)/total assets
Net input *Z*	z1	Off-balance sheet activity	Value of off-balance sheet activity
	z2	Equity	Book value of bank equity

Notes: Table shows the specific input and output variables used in efficiency estimation.

The literature suggests that bank efficiency can be calculated using either a non-parametric method (i.e., DEA) or a parametric approach (i.e., stochastic frontier analysis, SFA). We employ the most widely utilized methodologies, the stochastic frontier approach [29, 30] to gauge the efficiency when banks create the same liquidity under the same conditions, which not only posits specific functional forms for the efficiency frontier but also facilitates validity tests for estimated parameters. Building on the strengths of panel data, Greene [9] incorporates fixed effects within a frontier estimation framework. Drawing on his enhancements, the study specifies the stochastic frontier function for liquidity creation by banks in a panel dataset as follows:

$$\begin{array}{c} logQ_{i,t}=f_{Q}\left(W,Y,Z\right)+{\text{loga}}_{i}+{\text{logv}}_{i,t}\pm {\text{logu}}_{i,t} \end{array}$$ (1)

where *Q* represents cost or profit incurred. *f* is the cost or profit function. **Z** and **W** denote inputs and their price. **Y** is output and *a*_*i*_ is the bank-fixed effect. *u*_*i*,*t*_ ∼ *G* is the inefficiency factor and $v_{i,t}∼iid.N\left(0,\sigma_{v}^{2}\right)$ is random error. We set output as the bank’s liquidity creation level. As previously mentioned, liquidity creation offers a more complete and accurate representation of how banks serve the economy. Banking liquidity creation activities, like loans [31] and off-balance sheet activities [32, 33], boost economic development. Based on this model, Li et al. [22] compared the liquidity creation efficiency of global banks in time and space dimensions. With the distribution of *v* and *u* given, the inefficiency factor for the single bank can be estimated by maximum likelihood. Drawing on Greene’s [9]estimation approach, the expectation of inefficiency term can be calculated as:

$$\begin{array}{c} E\left(\mu_{i,t}|\varepsilon_{i,t}\right)=u_{*}+\sigma_{*}\frac{\phi (u_{*}/\sigma_{*})}{1-\Phi (u_{*}/\sigma_{*})} \end{array}$$ (2)

where $\sigma^{2}≔\sigma_{u}^{2}+\sigma_{v}^{2},u_{*}≔\sigma_{u}^{2}\varepsilon_{i,t}/\sigma^{2},\sigma_{*}^{2}≔\sigma_{u}^{2}\sigma_{v}^{2}/\sigma^{2},\lambda ≔\sigma_{u}/\sigma_{v}\phi ,u_{*}/\sigma_{*}=\varepsilon \lambda /\sigma .$ *ϕ* and Φ are the probability density and cumulative distribution functions of normal distributions, respectively. The formula can be reduced as:

$$\begin{array}{c} E\left(\mu_{i,t}|\varepsilon_{i,t}\right)=\sigma_{*}[\frac{\phi (\varepsilon_{i,t}\lambda /\sigma )}{1-\Phi (\varepsilon_{i,t}\lambda /\sigma )}+\frac{\lambda }{\sigma }\varepsilon_{i,t}] \end{array}$$ (3)

In line with the intrinsic goals of cost minimization and profit maximization in a bank’s operational processes, we distinguish between liquidity creation cost efficiency and liquidity creation profit efficiency. The assessment involves measuring the proximity of a bank’s generated profits or costs, given the prices of input factors, the output of liquidity creation, and other variables, to the efficient frontier represented by the banks with maximum profits or minimum costs. The bank liquidity creation cost efficiency (LCCE) can be measured as the ratio of a bank’s actual cost for creating the same level of liquidity under the same conditions to the minimum cost (best practice) a bank would incur. This efficiency measurement reflects the degree of efficiency with which a bank operates relative to the best-performing institutions within the sector.

$$\begin{array}{c} LCCE_{i}=\frac{C^{min}}{C_{i}}=\frac{\text{exp}[f(x_{i,t}\beta )]\text{exp}(\text{ln}\;v_{i,t})}{\text{exp}[f(x_{i,t}\beta )]\text{exp}(lnv_{i,t})\text{exp}(\text{ln}\;u_{i,t})}=\text{exp}(-\text{ln}\;{\hat{u}}_{i}^{C}) \end{array}$$

Similarly, we measure bank liquidity creation profit efficiency (LCPE) as the ratio of a bank’s actual profit to the profit a best-practice bank would attain.

$$\begin{array}{c} LCPE_{i}=\frac{P_{i}}{P^{max}}=\frac{\text{exp}[f(x_{i,t}\;\beta )]\text{exp}(lnv_{i,t})\text{exp}(-\text{ln}\;u_{i,t})}{\text{exp}[f(x_{i,t}\;\beta )]\text{exp}(\text{ln}\;v_{i,t})}=\text{exp}(-\text{ln}\;{\hat{u}}_{i}^{P}) \end{array}$$

where ${\hat{u}}_{i}^{C}$ and ${\hat{u}}_{i}^{C}$ denote the estimated inefficiency factor regarding cost and profit.

Then we specification of Bank liquidity creation cost and profit functions. The traditional Cobb-Douglas production function, characterized by substitution elasticity of one and constant returns to scale, poses challenges in replicating the true frontier of bank production processes. On the contrary, the transcendental logarithmic (translog) production function is extensively employed in panel estimations of the effects of bank efficiency on bank performance. In addition, many researchers found that it fits banks’ data better than other functional forms. Drawing on existing research [29, 30, 34], we specify cost and profit functions as follows:

$$\begin{array}{c} \begin{array}{cc} \text{ln}(\frac{Q}{w_{1}z_{2}})=\alpha_{0} & +\sum_{i=1}^{2}\alpha_{i}\;\text{ln}(w_{i}/w_{1})+\beta \;\text{ln}(LC/z_{2})+\gamma \;\text{ln}(z_{1}/z_{2})\\ & +\frac{1}{2}[\sum_{i=1}^{2}\sum_{j=1}^{2}\alpha_{ij}\;\text{ln}(w_{i}/w_{1})\text{ln}\;(w_{j}/w_{1})]\\ & +\beta_{11}\;\text{ln}(LC/z_{2})\text{ln}\;(LC/z_{2})+\gamma_{11}\;\text{ln}(z_{1}/z_{2})\text{ln}\;(z_{1}/z_{2})]\\ & +\sum_{i=1}^{2}\delta_{i1}\;\text{ln}(w_{i}/w_{1})\text{ln}\;(LC/z_{2})+\sum_{i=1}^{2}\zeta_{i1}\;\text{ln}(w_{i}/w_{1})\text{ln}\;(z_{1}/z_{2})+\\ & bX+\text{ln}\;u+\text{ln}\;v \end{array} \end{array}$$

where LC is liquidity creation defined in Section 3.1.1, denoting the bank system’s output. *w*_*i*_ and *z*_*i*_ are input prices and net inputs specified in Table 1, respectively. X includes control variables.

#### 3.1.3 Systemic risk and decomposition

We assess banks’ systemic risk by evaluating their sensitivity to shocks in the financial system, especially under distress events. The tail risk of individual financial institutions (IR) and the degree of connection with the financial system (SL) jointly determine the level of systemic risk. Therefore, we address such a distinction by decomposing the systemic risk estimator into two components to measure bank tail risk and systemic linkage, respectively.

This paper measures bank systemic through the method developed by van Oordt and Zhou [35]. The systemic risk of bank i is measured by the loss it experiences when an extremely adverse shock hits the financial system, namely, $\beta_{i}^{T}$ in the following tail model:

$$\begin{array}{c} R_{j}^{e}=\beta_{j}^{T}R_{m}^{e}+\varepsilon_{j},\;\text{for}\;R_{m}^{e}<-{\text{VaR}}_{m}\left(\bar{p}\right) \end{array}$$ (4)

where *R*_*i*_ and *R*_*s*_ denote the stock return of bank i and the financial system s, ${\text{VaR}}_{m}\left(\bar{p}\right)$ is the value-at-risk of the system. $\bar{p}$ notes a very small probability. With k worst ones out of n observations on the pair (*R*_*i*_, *R*_*s*_), *p* = *k*/*n*. The evaluation of $\beta_{i}^{T}$ is useful to assess the extreme loss on the stock in the event of a market crash. Van Oordt and Zhou [35] evaluated the potential drawbacks and applicable limitations of several kinds of measure methods for estimating $\beta_{i}^{T}$ and proposed an alternative estimator based on Extreme Value Theory(EVT).

Assuming *R*_*s*_ and *R*_*i*_ are both heavy-tailed, which can be expressed as

$$\begin{array}{c} \text{Pr}(R_{i}<-u)∼u^{-{\hat{\zeta }}_{i}}l_{i}\left(u\right)\;\text{and}\;\text{Pr}(R_{s}<-u)∼u^{-{\hat{\zeta }}_{s}}l_{s}\left(u\right)\;\text{as}\;u\to \infty \text{,}\; \end{array}$$

where *l*_*i*_(*u*), *l*_*s*_(*u*) are slowly varying functions as *u* → ∞

$$\begin{array}{c} \underset{u\to \infty }{\text{lim}\;}\frac{l_{i}\left(tu\right)}{l_{i}\left(u\right)}=\underset{u\to \infty }{\text{lim}\;}\frac{l_{s}\left(tu\right)}{l_{s}\left(u\right)}=1\;\text{for}\;\text{any}\;t>0 \end{array}$$

Then, we can consider the tail dependence measure using the EVT approach.

$$\begin{array}{c} \underset{p\to 0}{\text{lim}\;}{\left(\tau \left(p\right)\right)}^{\frac{1}{\xi_{s}}}\frac{VaR_{i}\left(p\right)}{VaR_{s(p)}}=\beta^{T} \end{array}$$ (5)

Eq 5 provides the basis of estimation of coefficient *β*^*T*^. For statistic estimation, we mimic the limit of the extreme value of *p* → 0 by focusing on the lowest observations of the tail region, denoted as k. The probability p can be rewritten as *p* = *k*/*n*, where *k*/*n* → 0 as *n* → ∞. $\hat{\zeta_{s}}$ is the tail index estimator proposed by Hill [36], $\hat{\tau (k/n)}$ is the nonparametric estimator of *τ*_*i*_ ≔ lim_*p* → 0_*τ*_*i*_(*p*), and can be estimated nonparametrically as:

$$\begin{array}{c} \tau_{i}\hat{\left(k/n\right)}≔\frac{1}{k}\sum_{t=1}^{n}\;I_{\left\{X_{t}^{\left(i\right)}>X_{n,n-k}^{\left(i\right)}\;\text{and}\;X_{t}^{\left(s\right)}>X_{n,n-k}^{\left(s\right)}\right\}} \end{array}$$

where the losses $X_{t}^{\left(m\right)}=-R_{m}\;\text{for}\;t=1,\ldots n$, and *I*_{}_ denotes a indicator function.The biggest advantage of this measure is that systemic risk measure *β*^*T*^ can be expressed and decomposed logarithmically into two conceptual components:

$$\begin{array}{c} \text{log}{\hat{\beta }}_{i}^{T}=\text{log}{\hat{\tau }}_{i}{\left(k/n\right)}^{1/{\hat{\zeta }}_{s}}+\text{log}\frac{{\hat{\text{VaR}}}_{i}\left(k/n\right)}{{\hat{\text{VaR}}}_{s}\left(k/n\right)}=:{\text{logSL}}_{i}+{\text{logIR}}_{i} \end{array}$$ (6)

Component *IR*_*i*_ (individual tail risk) captures the level of bank tail risk. Since the denominator *VaR*_*s*_(*p*) is homogeneous across all financial institutions, the cross-sectional variation in *IR*_*i*_ is solely due to the variation in the tail risks of individual banks, *VaR*_*i*_(*p*). Component *SL*_*i*_ (systematic linkage) measures the strength of the link between the bank and the system in financial distress. Since *τ*_*i*_(*p*) is unaffected by any strictly increasing monotonic transformation of *R*_*i*_, cross-sectional differences in *SL*_*i*_ contain information only on the dependence between extreme shocks in the financial system and severe losses suffered by a particular bank, without being affected by the level of bank tail risk. As the sum of individual tail risk and systemic linkage, $\beta_{i}^{T}$(beta) is systemic risk.

We use the return of the China Shenwan Banking Industry Index to proxy financial system return *R*_*s*_, and set *k*/*n* ≈ 5%, similar to values in previous studies. Our baseline results fix a rolling estimation window of 8-quarter daily return for each bank.

**Fig 2:**
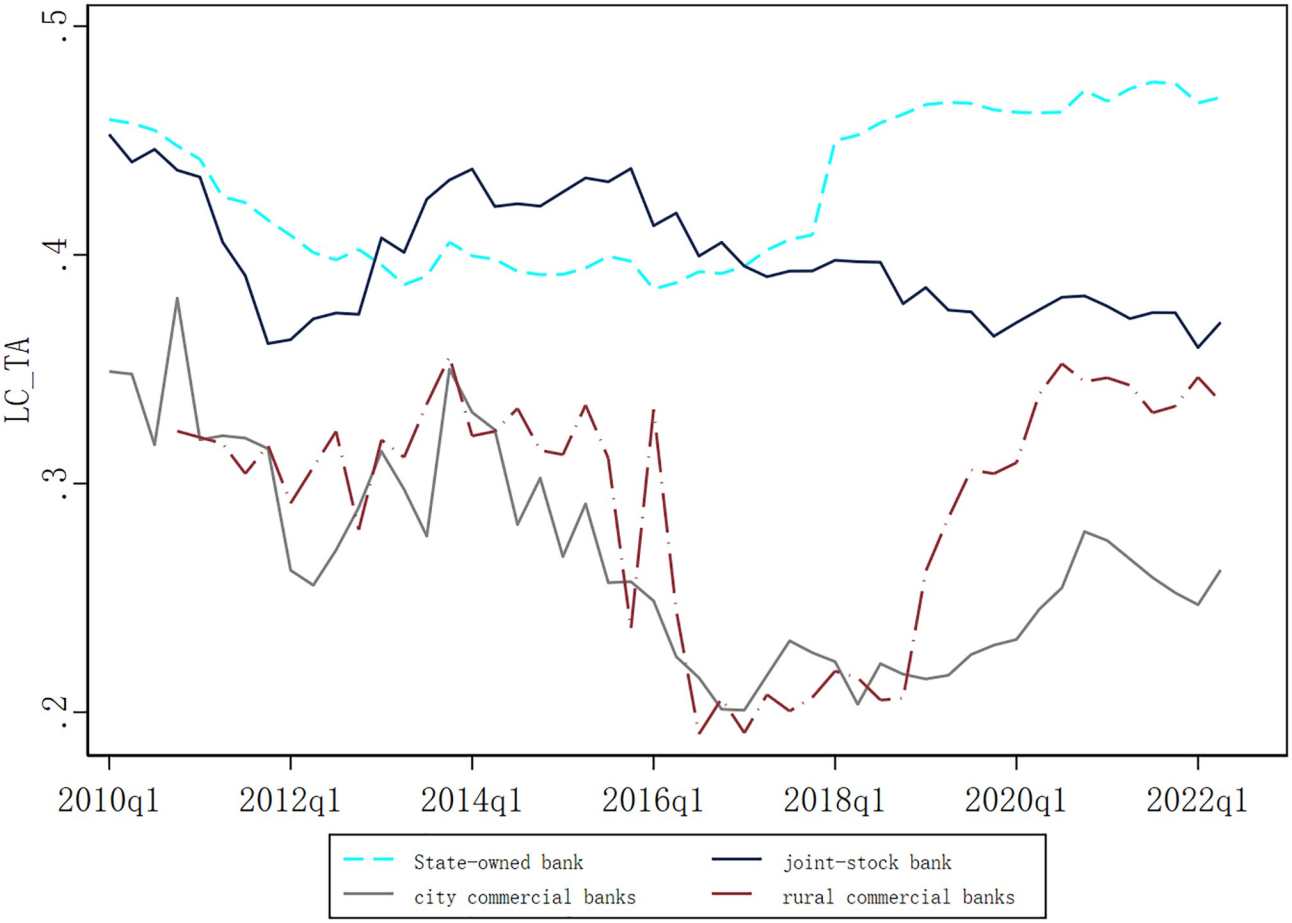
Decomposition of bank systemic risk in China. This figure plots quarterly estimation results of total systematic risk(beta), individual tail risk(IR), and systematic linkage(SL) between sample periods. These three categories are defined in Section 3.1.3.

The risk decomposition results for China’s bank sample are presented in Fig 2. The total systematic risk (beta) measure highlights several significant events in the Chinese market, such as the 2015 stock market crash, the 2008 global financial crisis, and the 2005 non-tradable shares reform. Beta rose sharply before these crashes and then plummeted. There was generally a negative correlation between IR and SL, particularly during crises where IR usually increases and SL decreases. This implies that banks suffered severe adverse tail shocks and became disconnected from the banking system. SL tends to be at a relatively high level before a crisis. Its decline is steep when a crisis occurs, although its volatility is relatively small during normal times.The individual tail risk of the bank and the risk of systematic linkage are in a state of mutual transformation. Before the crisis, banks were usually in a state of high linkage. However, during the crisis, the reasons for the decline in bank linkage are as follows. First, banks will fire sell assets to reduce indirect connection. Second, the shock has been transformed into an individual due to network contagion, which is reflected in an individual tail risk. This finding reflects the paradoxical robustness-while-fragile characteristic of the Chinese banking system. The distinct patterns of the two components also validate the advantages of decomposing the risk.

#### 3.1.4 Other bank characteristics and summary statistics

To further mitigate the confounding effects of omitted variables on the results, this paperincorporates variables that reflect the latent risks in the banking operational process as control variables. The scale of a bank (Size) is closely correlated with its efficiency, whereby business scale may encompass additional risks, yet conversely, it could diminish overall risk due to risk diversification. Capital ratio (Capr) reflects the bank’s leverage level, and operational volatility in banking may be influenced by leverage amplification. Net return on equity (ROE) and non-performing loan ratio (NPL) directly depict the bank’s profitability and default risk in its primary lending operations. Meanwhile, the non-interest income ratio reflects the diversification trend in banking operations. Non-interest income (NII) may simultaneously enhance a bank’s profit potential and augment its sources of risk. Table 2 presents the summary statistics of key variables.

**Table 2:** Summary statistics of key variables.

VarName	Definition	Description	Source
lnSL	Systematic linkage	estimated in 3.1.3	Authors’ calculations using data from Wind
lnIR	Individual risk	estimated in 3.1.3	Authors’ calculations using data from Wind
lnbeta	Systematic risk	estimated in 3.1.3	Authors’ calculations using data from Wind
LCPE	Profit efficiency of LC	estimated in 3.1.2	Authors’ calculations using data from Wind
LCCE	Cost efficiency of LC	estimated in 3.1.2	Authors’ calculations using data from Wind
Size	total assets	ln(total assets)	China Stock Market and Accounting Research Database
Capr	Capital ratio	total equity/ total assets	China Stock Market and Accounting Research Database
ROE	Net return on equity	net income/total euqity	China Stock Market and Accounting Research Database
NII	Non-interest income ratio	non-interest income/operating income	China Stock Market and Accounting Research Database
NPL	Non-performing loan ratio	non-performing loan/total loan	China Stock Market and Accounting Research Database

VarName	Obs	Mean	SD	Min	Median	Max
lnSL	1544	-0.22	0.14	-0.85	-0.18	-0.06
lnIR	1544	0.19	0.36	-0.66	0.18	1.51
lnbeta	1544	-0.03	0.31	-0.92	0.01	0.80
LCPE	1423	0.85	0.21	0.04	0.90	1.00
LCCE	1424	0.16	0.24	0.00	0.06	1.00
Size	1947	27.76	1.73	24.18	27.73	31.04
Capr	1940	6.51	1.68	2.01	6.62	10.00
ROE	1954	9.84	5.30	2.05	9.04	25.83
NII	1896	20.57	10.79	0.76	19.64	46.68
NPL	1843	1.63	1.56	0.36	1.33	11.41

Notes: The table shows descriptive statistics for dependent variables, independent variables, and control variables.lnSL,lnIR, and lnbeta are systematic risk and its components defined in Section 3.1.3. LCPE and LCCE are bank liquidity creation cost/profit efficiency defined in Section 3.1.2. Size is the logarithm of total bank assets. Capr is the bank capital ratio. ROE is the net return on equity. NII is non-interest income divided by operating income. NPL is the non-performing loan ratio. The sample period is January 2000 to January 2021.

In China, banks are typically categorized based on their equity structures into state-owned banks, joint-stock banks, city commercial banks, and rural commercial banks. These categories of banks exhibit significant differences in their business scope, funding costs, operational models, and efficiency objectives. Consequently, we conduct a deeply descriptive statistical analysis of the profit efficiency and cost efficiency for each type of bank. The results are presented in Table 3.

**Table 3:** Descriptive statistical summary of liquidity creation efficiency measure.

VarName	Obs	Mean	SD	Min	Median	Max
**State**						
LCPE	303	0.95	0.17	0.03	1.00	1.00
LCCE	304	0.03	0.10	0.00	0.01	1.00
**Joint-stock banks**						
LCPE	476	0.91	0.19	0.05	1.00	1.00
LCCE	476	0.04	0.05	0.00	0.03	0.25
**Rural and city Commercial Bank**						
LCPE	644	0.91	0.22	0.03	1.00	1.00
LCCE	644	0.31	0.29	0.02	0.22	1.00

Notes: This table compiles descriptive statistics for cost efficiency and profit efficiency of liquidity creation, categorized by different bank ownership structures. Cost efficiency(LCCE) and profit efficiency(LCPE) of liquidity creation are defined in Section 3.1.2. The sample period is January 2000 to January 2021.

Profit efficiency among Chinese banks does not exhibit significant variation overall, whereas there are notable discrepancies in cost efficiency. Banks with the highest profit efficiency are predominantly state-owned, reflecting their market share dominance and relatively superior profitability. On the other hand, rural commercial banks and city commercial banks demonstrate higher cost efficiency, respectively. Despite lacking a cost advantage in terms of capital, these banks have proactively advanced unit cost output. Banks are becoming probability through methods such as project management to establish a competitive edge in cost control.

### 3.2 Data description

This paper collects panel data for a sample of China A-share listed banks over the period 2000–2021 from the China Stock Market & Accounting Research Database (CSMAR) and the Wind database. We have also manually supplemented some variable observations from bank annual reports. We have winsorized all continuous variables at the 1st and 99th percentiles to ensure consistency.

### 3.3 Empirical methodology

To explore the empirical relationship between systemic risk and bank liquidity creation efficiency, we estimate three regression models using our estimates of systemic risk(*lnbeta*), systemic linkage(*lnSL*), and bank tail risk(*lnIR*) as dependent variables, respectively. We estimate the coefficients in the following models from panel data.

$$\begin{array}{c} \begin{array}{cc} lnbeta_{i,t} & =\alpha +\beta LCE_{i,t-1}+\gamma X_{i,t-1}+\theta_{t}+\delta_{i}+v_{i,t}\\ lnSL_{i,t} & =\alpha +\beta LCE_{i,t-1}+\gamma X_{i,t-1}+\theta_{t}+\delta_{i}+\epsilon_{i,t}\\ lnIR_{i,t} & =\alpha +\beta LCE_{i,t-1}+\gamma X_{i,t-1}+\theta_{t}+\delta_{i}+e_{i,t} \end{array} \end{array}$$ (7)

Where *θ*_*t*_ is time-fixed effects, capturing variation in macroeconomic state variables and other sources of common variation in systemic risk over time and *δ*_*i*_ is bank fixed effects, capturing characteristics of a bank that do not change over time. The bank individual fixed effect and the time fixed effect are used to alleviate the interference of the important explanatory variable omission. Independent variable *LCE*_*i*,*t*−1_ is lagged bank liquidity creation efficiency, including profit and cost efficiency defined previously. We follow the related literature [4, 37] and control a set of bank characteristics *X*_*i*,*t*_. Finally, *v*_*i*,*t*_, *ϵ*_*i*,*t*_, *e*_*i*,*t*_ are the error terms respectively.

## 4 Empirical results

### 4.1 The double-edge effect of efficiency

Table 4 presents the empirical results of the Eq 7. Columns (1)-(3) show the double-edged effect of bank liquidity creation profit efficiency: Specifically, while there is a notable negative correlation between bank liquidity creation profit efficiency (LCPE) and individual risk (IR), the enhancement of LCPE is also linked to an increase in system linkage (SL), suggesting a possible trade-off between these two effects. The total impact of bank profit efficiency on systemic risk is negative, indicating the dominant role of individual risk (IR). The t-statistic of *β*_1_ is -3.92, which means coefficient *β*_1_ is statistically less than zero. The regression results support Hypothesis 1 which proposes that Bank liquidity creation efficiency will reduce the bank’s overall systematic risk. Despite the current lack of observable adverse impacts of liquidity creation profit efficiency on the overall systemic risk from the data, this may lead to an underappreciation of the inherent risks associated with increased systemic interconnectedness. Particularly in China, banks enhance their profit efficiency through non-traditional transactions among interbank or non-banking financial institutions, which increases their systematic linkage to the system. However, the potential risks accumulated related to systematic linkage are often overlooked, with attention solely focused on the changes in aggregate risk. It is noteworthy that efficiency improvement embodies a double-edged sword effect. Reducing individual risks while increasing the interconnectedness of the financial system may finally increase the risk of contagion.

**Table 4:** The relationship between bank liquidity creation efficiency and systemic risk.

	(1)	(2)	(3)	(4)	(5)	(6)	(7)	(8)	(9)
	lnSL	lnIR	lnbeta	lnSL	lnIR	lnbeta	lnSL	lnIR	lnbeta
LCPE	0.070***	-0.184***	-0.098**				0.068***	-0.181***	-0.098**
	(0.018)	(0.043)	(0.043)				(0.018)	(0.043)	(0.044)
LCCE				0.043	-0.072	-0.021	0.026	-0.026	0.003
				(0.028)	(0.067)	(0.067)	(0.028)	(0.067)	(0.068)
Size	0.036*	-0.004	0.036	0.053***	-0.036	0.024	0.044**	-0.012	0.037
	(0.019)	(0.045)	(0.045)	(0.020)	(0.049)	(0.049)	(0.020)	(0.049)	(0.050)
Capr	0.007*	0.032***	0.039***	0.006*	0.034***	0.040***	0.007*	0.032***	0.039***
	(0.004)	(0.009)	(0.009)	(0.004)	(0.009)	(0.009)	(0.004)	(0.009)	(0.009)
ROE	-0.001**	0.004**	0.002	-0.001	0.002	0.001	-0.001**	0.004**	0.002
	(0.001)	(0.002)	(0.002)	(0.001)	(0.002)	(0.001)	(0.001)	(0.002)	(0.002)
NII	0.001	0.004***	0.005***	0.001	0.004***	0.005***	0.001	0.004***	0.005***
	(0.000)	(0.001)	(0.001)	(0.000)	(0.001)	(0.001)	(0.000)	(0.001)	(0.001)
NPL	-0.024***	-0.085***	-0.111***	-0.028***	-0.078***	-0.108***	-0.026***	-0.084***	-0.111***
	(0.007)	(0.017)	(0.017)	(0.007)	(0.017)	(0.017)	(0.007)	(0.017)	(0.017)
Constant	-1.326**	0.210	-1.238	-1.735***	0.933	-0.996	-1.548***	0.432	-1.267
	(0.530)	(1.284)	(1.289)	(0.581)	(1.411)	(1.409)	(0.580)	(1.406)	(1.412)
Bank_FE	Yes	Yes	Yes	Yes	Yes	Yes	Yes	Yes	Yes
Year_FE	Yes	Yes	Yes	Yes	Yes	Yes	Yes	Yes	Yes
Observations	1207	1207	1207	1207	1207	1207	1207	1207	1207
Adjusted *R*^2^	0.535	0.658	0.617	0.529	0.653	0.616	0.535	0.658	0.617

Notes: This table reports the estimated regression coefficients of the linear regression in Eq 7. The dependent variable is a systematic risk(ln beta) and its components(ln SL,lnIR). The independent variables are bank liquidity creation profit efficiency(LCPE) and bank liquidity creation cost efficiency(LCCE). Variable Size is defined as a log asset. Capr is defined as the ratio of equity to total assets. ROE is the return on equity. NII is non-interest income divided by operating income. NPL is the non-performing loan ratio. Both LCPE and LCCE are controlled in the last three columns to ensure robustness. The sample starts in January 2000 and ends in January 2021.

*, ** and *** indicate statistical significance at the 10%, 5% and 1% levels, respectively. Values in parentheses are standard errors clustered at bank level.

Columns (4)-(6) show that bank liquidity creation cost efficiency (LCCE) is not significantly related to risk. This supports the view that LCPE and LCCE reflect different abilities of banks, rendering the correlation between LCPE and LCCE indefinite [5]. Our findings for Chinese banks differ from U.S. banks, whose cost efficiency significantly correlates to risk rather than profit efficiency [4]. In the course of cost control, Chinese banks have not accumulated risk per se. Instead, it is in the pursuit of profit that they have heightened their interconnectedness with the financial system, thereby potentially precipitating a crisis of systemic contagion. This heightened linkage, while ostensibly fostering efficiency to profit maximization, inadvertently augments the financial system’s vulnerability to the propagation of risk. Consequently, a failure within one institution can cascade through the network, engendering a domino effect that threatens the entire system’s stability. This phenomenon underscores the need for vigilant regulatory oversight of bank behaviors engaging in profit chasing. Columns (7)-(9) incorporate both profit and cost efficiency into the regression model while controlling for potential mutual influences. The results remain unaffected. The relationship between cost efficiency and risk is insignificant, whereas the impact of profit efficiency on risk continues to exhibit a double-edged sword effect.

Theoretically, there are contradicting channels through which bank efficiency affects risk. High-cost efficiency may reflect superior managerial quality, resulting in favorable performance [26, 38], or indicate “skimping” on resources to screen and monitor loan applicants, leading to poor loan outcomes that become apparent only during subsequent financial crisis [26]. High-profit efficiency may be associated with high charter values that produce favorable performance [39, 40], or excessive risk-taking that earns high returns in normal times but creates problems in subsequent crises [4]. Thus, a natural question arises: does the relationship between bank liquidity creation efficiency and risk in China, diverging from the conclusions of existing literature, reflect a different story? We explore the answer by delving deeper into the channels through which bank efficiency influences risk.

### 4.2 Bank liquidity creation efficiency, managerial quality, and risk-taking

We employed the principal component analysis to construct a measure reflecting the managerial quality of banks. Based on relevant findings in corporate governance research [41], we selected indicators across six major categories: ownership structure indicators (including the shareholding ratio of the largest shareholder, the ratio of the shareholding by the second to fifth largest shareholders to that of the largest shareholder), nature of property indicator (a binary variable indicating whether the largest shareholder is a state-owned shareholder), controlling shareholder behavior indicators (the ratio of related transactions measured as the sum of sales to and purchases from related parties divided by total assets at the end of the period), management governance indicators (a binary variable indicating whether the company Chairman and CEO concurrently serve), board governance indicators (board size, proportion of independent directors), and external market competition indicators (market share measured as the company’s operating revenue divided by the total operating revenue of the industry). These indicators were subjected to principal component analysis, with the score of the first principal component serving as the indicator reflecting the managerial quality [42].

Concerning the measurement of risk-taking on the asset side of banks, the literature commonly employs indicators such as non-performing loan ratios and Z-scores. However, these variables essentially gauge banks’ post facto risk-taking behavior. To assess banks’ ex-ante risk-taking behavior, this study elected the proportion of risk-weighted assets to total assets as a measure.

We conduct panel regressions similar to the model 7, except that the dependent variables are replaced by the corresponding channel variables, namely managerial quality or risk-taking. Columns (1)-(3) in Table 5 show that the improvement in bank liquidity creation efficiency is not correlated with the improvement in managerial quality in China. This differs from the evidence that bank cost efficiency is associated with managerial quality in the U.S. [4], which implies that banks in China may improve liquidity creation cost efficiency by devoting fewer resources to credit screening and monitoring [26]. However, higher bank liquidity creation profit efficiency in China is accompanied by more risk-taking on loans, as Table 5 Columns (4)-(6) show. The empirical results support hypothesis 3 (high risk-taking appetize hypothesis) rather than hypothesis 2 (high management level hypothesis). Thus, an interesting question is: what loan structure do banks in China look like?

**Table 5:** Bank liquidity creation efficiency, bank managerial quality, and risk-taking.

	Managerial quality			Risk taking		
	(1)	(2)	(3)	(4)	(5)	(6)
LCCE	-0.122		-0.120	-0.047		-0.061*
	(0.111)		(0.113)	(0.032)		(0.032)
LCPE		-0.027	-0.011		0.054**	0.061***
		(0.080)	(0.081)		(0.022)	(0.022)
Size	-0.923***	-0.884***	-0.922***	0.302***	0.313***	0.294***
	(0.087)	(0.080)	(0.088)	(0.024)	(0.022)	(0.024)
Capr	-0.124***	-0.125***	-0.124***	-0.003	-0.003	-0.003
	(0.015)	(0.015)	(0.015)	(0.004)	(0.004)	(0.004)
ROE	0.007***	0.008***	0.008***	-0.003***	-0.003***	-0.003***
	(0.003)	(0.003)	(0.003)	(0.001)	(0.001)	(0.001)
NII	0.000	0.000	0.000	-0.001*	-0.001	-0.001
	(0.002)	(0.002)	(0.002)	(0.001)	(0.001)	(0.001)
NPL	0.046	0.038	0.046	0.014*	0.012	0.015*
	(0.030)	(0.029)	(0.030)	(0.008)	(0.008)	(0.008)
Constant	25.280***	24.183***	25.249***	-7.203***	-7.561***	-7.026***
	(2.495)	(2.295)	(2.507)	(0.686)	(0.629)	(0.687)
Bank_FE	Yes	Yes	Yes	Yes	Yes	Yes
Year_FE	Yes	Yes	Yes	Yes	Yes	Yes
Observations	1077	1077	1077	1179	1179	1179
Adjusted *R*^2^	0.753	0.752	0.752	0.877	0.877	0.878

Notes: This table presents the results of channel searching. The managerial quality of a bank is measured by the first principal component of six categories. Risk-taking is measured by the ratio of risk-weighted assets to loans.

*, ** and *** indicate statistical significance at the 10%, 5% and 1% levels, respectively. Values in parentheses are standard errors clustered at bank level.

### 4.3 Bank liquidity creation profit efficiency and allocation of loan

China’s real estate industry experienced rapid and sustained development and acted as a significant contributor to the country’s economic growth. According to the China National Bureau of Statistics, real estate investment has accounted for more than 10% of GDP since 2009. The manufacturing industry is usually a proxy for the real sector and the key to industrial upgrading in China. We empirically explore the relationship between bank liquidity creation profit efficiency and loan allocation to these two industries. As is shown in Tables 6 and 7, the high liquidity creation profit efficiency of banks in China is closely associated with more investment in the real estate industry and less investment in the manufacturing industry, which implies that funds may be diverted away from the manufacturing sector. Banks played a crucial role in the real estate industry development and made steady and profitable returns, thereby enhancing their profit efficiency and helping alleviate individual tail risk, IR. However, as highlighted by Allen and Gale [43], the flow of a large amount of money from the banking industry into the real estate market creates a complex network between the banking and real estate industry, resulting in overlapping closed ties among banks and increasing the potential for financial contagion and systemic risk. This affects not only the link between banks and real estate firms but also the connection between banks themselves due to the concentration of credit resources in the real estate sector. The debt problems originated from Evergrande (a famous real estate enterprise in China) in 2021, which is a good example. Therefore, while increased liquidity creation profit efficiency may decrease individual tail risk IR, it also leads to a rise in systemic linkage, SL, which may result in the accumulation of bubbles and future risks. This echoes the double-edged effect of bank liquidity creation efficiency.

**Table 6:** The bank liquidity efficiency and bank loans allocation.

$\frac{loanstoindustry}{totalloans}$ %	(1)	(2)	(3)
	High PE	Low PE	(1)-(2)
Real estate	12.08	9.83	2.25***
	(5.03)	(5.24)	(7.54)
Manufacturing	19.27	26.87	-7.60***
	(10.65)	(11.94)	(12.95)

Notes: Banks are divided into two groups based on whether their liquidity creation profit efficiency is higher than the median level. A two-sample t-test is conducted to indicate whether banks in High PE groups allocate significantly more loans to the real estate industry on average. Values in parentheses of columns (1)-(2) are standard deviations. Values in parentheses of column (3) are t-statistics.

**Table 7:** The bank liquidity efficiency and bank loans allocation.

	Managerial quality			Real estate		
	(1)	(2)	(3)	(4)	(5)	(6)
Lag LCCE	-0.047***		-0.050***	0.052***		0.051***
	(0.011)		(0.011)	(0.009)		(0.009)
Lag LCPE		-0.008	-0.007		0.018***	0.017***
		(0.007)	(0.007)		(0.006)	(0.006)
Size	-0.020**	-0.002	-0.020**	0.050***	0.030***	0.049***
	(0.009)	(0.008)	(0.009)	(0.007)	(0.007)	(0.007)
Capr	0.002	0.000	0.001	0.007***	0.008***	0.007***
	(0.002)	(0.002)	(0.002)	(0.001)	(0.001)	(0.001)
ROE	-0.000	-0.000*	-0.000	-0.000	-0.000	-0.000
	(0.000)	(0.000)	(0.000)	(0.000)	(0.000)	(0.000)
NII	0.000	0.000	0.000	0.000	0.000	0.000
	(0.000)	(0.000)	(0.000)	(0.000)	(0.000)	(0.000)
NPL	0.006*	0.006*	0.010***	0.012***	0.018***	0.014***
	(0.004)	(0.004)	(0.004)	(0.003)	(0.003)	(0.003)
Constant	0.772***	0.273	0.789***	-1.375***	-0.837***	-1.366***
	(0.263)	(0.230)	(0.256)	(0.210)	(0.188)	(0.208)
Bank_FE	Yes	Yes	Yes	Yes	Yes	Yes
Year_FE	Yes	Yes	Yes	Yes	Yes	Yes
Observations	1169	1168	1168	1169	1168	1168
Adjusted *R*^2^	0.911	0.914	0.915	0.770	0.767	0.773

Notes: This table represents the results of channel searching. The explained variable manufacturing means manufacturing loans as a percentage of total loans. Real estate is the percentage of total bank loans allocated to real estate.

*, ** and *** indicate statistical significance at the 10%, 5% and 1% levels, respectively. Values in parentheses are standard errors clustered at bank level.

## 5 Endogeneity and robustness check

In this paper, the potential reverse causality in endogeneity issues appears to be limited. The independent variable, liquidity creation efficiency, is based on contemporary data, while the dependent variable, systemic risk in the banking sector, is derived from calculations within a subsequent event window. Consequently, the time series analysis indicates a minimal likelihood of future events significantly influencing the current period. Nevertheless, there may be omitted variables in the model problems, especially the individual characteristics of banks that do not change over time. Therefore, in this paper, the bank fixed effect is controlled for testing. Liquidity creation profit efficiency still significantly reduces individual effects but increases the connectivity of individual banks to the system.

This study conducts a series of robustness tests.

(1) Exclude the extreme impacts of stock market crises.

Since the systemic risk indicator construction is based on stock market data. In both 2015 and 2007, the A-share market witnessed sustained periods of significant negative returns. In order to mitigate the potential influence of extreme market sentiments on empirical results, this section excludes samples corresponding to the aforementioned extreme return intervals. The results are presented in Table 8, and the main results are robust.

**Table 8:** Robustness check of excluding the extreme impacts from stock market crises.

	(1)	(2)	(3)
	lnSL	lnIR	lnbeta
LCPE	0.053**	-0.140***	-0.087*
	(0.022)	(0.052)	(0.053)
Size	0.079***	0.106**	0.185***
	(0.019)	(0.045)	(0.046)
Capr	0.014***	0.063***	0.077***
	(0.004)	(0.011)	(0.011)
ROE	-0.001*	-0.001	-0.002
	(0.001)	(0.002)	(0.002)
NII	0.000	0.004***	0.004***
	(0.001)	(0.001)	(0.001)
NPL	-0.061***	-0.197***	-0.258***
	(0.012)	(0.028)	(0.028)
Constant	-2.528***	-2.979**	-5.507***
	(0.540)	(1.295)	(1.314)
Bank_FE	Yes	Yes	Yes
Year_FE	Yes	Yes	Yes
Observations	1098	1098	1098
Adjusted R2	0.535	0.672	0.642

Notes: This table presents the robustness check results of model 7. We exclude the extreme impacts of stock market crises in 2015 and 2007 and conduct robust tests on the main regression. The dependent variables are systematic risk(lnbeta) and its components (lnSL, lnIR). The independent variable is bank liquidity creation profit efficiency(LCPE) or bank liquidity creation cost efficiency(LCCE). Variable Size is defined as the logarithm of the asset. Capr is defined as the ratio of equity to total assets. ROE is the return on equity. NII is non-interest income divided by operating income. NPL is the non-performing loan ratio. The sample starts in January 2000 and ends in January 2021.

*, ** and *** indicate statistical significance at the 10%, 5% and 1% levels, respectively. Values in parentheses are standard errors clustered at bank level.

(2) Exclude samples with extreme risk.

Extreme values of the systemic risk for banks correspond to low probability events, potentially attributed to public sentiment, stock crises, unforeseen circumstances, or purely exogenous shocks. Such extreme situations may not be directly related to the internal operations of banks and could introduce disturbances to the regression results. In this section, we exclude samples where the systemic risk measure exceeds the 95th percentile. The results are still robust (Table 9).

**Table 9:** Robustness check of excluding extreme risk sample.

	(1)	(2)	(3)
	lnSL	lnIR	lnbeta
LCPE	0.079***	-0.163***	-0.084*
	(0.018)	(0.043)	(0.043)
Size	0.031	-0.022	0.009
	(0.019)	(0.046)	(0.046)
Capr	0.008**	0.028***	0.036***
	(0.004)	(0.009)	(0.009)
ROE	-0.002***	0.003**	0.002
	(0.001)	(0.002)	(0.002)
NII	0.000	0.003***	0.004***
	(0.000)	(0.001)	(0.001)
NPL	-0.026***	-0.091***	-0.117***
	(0.007)	(0.017)	(0.017)
Constant	-1.182**	0.748	-0.433
	(0.554)	(1.303)	(1.307)
Bank_FE	Yes	Yes	Yes
Year_FE	Yes	Yes	Yes
Observations	1190	1190	1190
Adjusted R2	0.531	0.632	0.587

Notes: This table presents the robustness check results of model 7. We exclude extreme samples of systematic risk and conduct robust tests on the main regression. The dependent variables are systematic risk(lnbeta) and its components (lnSL, lnIR). The independent variable is bank liquidity creation profit efficiency(LCPE) or bank liquidity creation cost efficiency(LCCE). Variable Size is defined as the logarithm of the asset. Capr is defined as the ratio of equity to total assets. ROE is the return on equity. NII is non-interest income divided by operating income. NPL is the non-performing loan ratio. The sample starts in January 2000 and ends in January 2021.

*, ** and *** indicate statistical significance at the 10%, 5% and 1% levels, respectively. Values in parentheses are standard errors clustered at bank level.

(3) The replacement of extreme probability in the tail distribution.

In estimating the parameter $\beta_{j}^{T}$ using extreme value theory, a small value of the parameter k will increase estimation uncertainty, while setting k relatively large may result in significant measurement error. Literature commonly adopts a range within 4% to 5% for the tail probability p(p = k/n). To assess the sensitivity of the conclusions to the choice of the tail probability p, the tail probability p is changed from 5% to 4% and then recalculates bank systematic risk. The robustness results of the main regression are demonstrated in Table 10, and the main conclusion is unchanged.

**Table 10:** Robustness check of replacing tail probability.

	(1)	(2)	(3)
	lnrSL	lnrIR	lnrbeta
LCPE	0.052***	-0.211***	-0.122***
	(0.018)	(0.046)	(0.046)
Size	0.018	0.011	0.068
	(0.019)	(0.049)	(0.048)
Capr	0.010***	0.032***	0.041***
	(0.004)	(0.009)	(0.009)
ROE	0.000	0.005***	0.003*
	(0.001)	(0.002)	(0.002)
NII	0.001**	0.004***	0.005***
	(0.000)	(0.001)	(0.001)
NPL	-0.032***	-0.077***	-0.106***
	(0.007)	(0.018)	(0.018)
Constant	-0.818	-0.201	-2.149
	(0.548)	(1.409)	(1.379)
Bank_FE	Yes	Yes	Yes
Year_FE	Yes	Yes	Yes
Observations	1199	1214	1199
Adjusted R2	0.518	0.634	0.606

Notes: This table presents the robustness check results of replacing tail probability. We change tail probability p from 5% to 4% and then recalculate bank systematic risk to conduct robust tests on the main regression. The main regressions are concluded using new systematic measurements. The dependent variables are systematic risk(lnbeta) and its components(lnSL,lnIR). The independent variable is bank liquidity creation profit efficiency(LCPE) or bank liquidity creation cost efficiency(LCCE). Variable Size is defined as the logarithm of the asset. Capr is defined as the ratio of equity to total assets. ROE is the return on equity. NII is non-interest income divided by operating income. NPL is the non-performing loan ratio. The sample starts in January 2000 and ends in January 2021.

*, ** and *** indicate statistical significance at the 10%, 5% and 1% levels, respectively. Values in parentheses are standard errors clustered at bank level.

## 6 Extensions: The role of digital transformation

The digital wave has already swept through the financial system, and digital transformation is widely believed to reduce costs by improving operational efficiency. Banks with good financial technology development have taken the lead in realizing the digital intelligence of their business and achieving strategic upgrading. Therefore, many studies have focused on the value of digital transformation on the cost side of banks. Digital transformation can change the working process of both cost minimization and profit maximization. In terms of market expansion, the operation of fintech by banks has dramatically enhanced the customer availability of banks. Regarding asset allocation, fintech can accurately match the risk gap and the capital gap, optimize the asset allocation, and thus obtain a new range of interest growth. In terms of risk control, using multidimensional and massive real business data, fintech banks can build intelligent loan evaluation models to assist business decisions and reduce bank losses caused by nonconforming rates. In general, banks with different degrees of digitalization have apparent differences in cost control and profit growth processes. The evolution of banking operations will ultimately manifest in variations in risk profiles.

The innovation growth view focuses on the bright side of financial innovation, positing that financial innovations improve the functions of the financial system as they reduce agency costs, foster risk sharing, and improve allocation efficiency [44]. Then, we verified whether there were differences in the relationship between efficiency and risk among banks at different levels of digital transformation according to the discussion above. The characteristic information related to the digital transformation of enterprises is more easily reflected in Annual reports of a summary and instructive nature. Therefore, the word frequency related to “digital transformation” in the bank’s annual report is counted to characterize it. Specifically, we conduct bank digital transformation level estimation referring to Wu Wenyang et al. [45]. Banks’ digital transformation is subdivided into “underlying technology” and “practical application”, which includes not only four typical digital transformation technologies but also the application performance of such technologies in specific practices. The specific classification dictionary is displayed in Table 11.

**Table 11:** The thesaurus of the digital transformation index for banks.

Digitization dimensions		Keyword lexicon
Underlying technology	Artificial intelligence	Artificial Intelligence, Business Intelligence, Image Understanding, Investment Decision Assistance System, Intelligent Data Analysis, Sharing, Machine Learning, Deep Learning, Semantic Search, Biometrics, Face Recognition, Speech Recognition, Identity Verification, Portraits, Precise Matching, Customization, Agility Robotics
	Blockchain	Blockchain, digital currency, distributed accounting, distributed computing, differential privacy technology, smart financial contracts
	Cloud computing	Cloud computing, stream computing, graph computing, in-memory computing, multi-party secure computing, brain-like computing, green computing, cognitive computing, converged architecture, 100 million concurrency, exabyte-level storage, IT, Internet of Things, cyber-physical systems, private cloud, public cloud
	Big data	Big Data, Digital Mining, Information Technology, Text Mining, Data Visualization, Heterogeneous Data, Credit Investigation, Augmented Reality, Mixed Reality, Virtual Reality, Data Analysis
Practical application	Digital application	Internet Finance, Mobile Banking, APP, NFC Payment, Mobile Payment, Mobile Payment, Third-party Payment, E-commerce, B2B, B2C, C2B, C2C, O2O, Networking, Platform, Intelligent Supervision, Smart Wearable, Smart Agriculture, Intelligent Risk Control, Intelligent Transportation, Smart Healthcare, Intelligent Customer Service, Intelligent Investment Advisory, Smart Counter, Regulatory Technology, Digital Marketing, Intra-city Communication, Real-time Remittance, Digital Finance, Fintech, Fintech, Fintech, Quantitative Finance, Open Banking, API, Online banking, private banking, scenarios, supply chain finance, and digital inclusive finance

Notes: This table is a summary of the keywords extracted from the bank’s annual report on digital transformation. We divide them into two categories: underlying technology and application technology. Among them, the underlying technology includes four typical specific digital transformation technologies, while the application technology involves the specific application of digital transformation technology in banks.

We divided Chinese banks into two groups according to the level of digital transformation estimated as above, below average, or above average. The results of conducting research model 7 on two subgroups are presented in Table 12. Overall, the relationship between the cost efficiency of liquidity creation and systemic risk of the two types of banks is not obvious, indicating that the average effect of the main test does not differ between the two groups. However, the relationship between liquidity profit efficiency and risk of the two types of banks is entirely opposite. The higher the level of digitalization, the higher the profit efficiency of banks, which not only will not improve the connectivity with the system but also help to reduce the correlation with the system, while the low digital transformation of banks, still in the process of improving profit efficiency, have to increase the connection with the entire system, resulting in the overall systemic risk increase. Network correlation refers to the state in which banks are closely connected through joint asset holding and inter-bank business. It can be inferred that the profit business of banks with a low degree of digital transformation is still more biased towards traditional interbank business. Digital transformation helps banks to carry out their own distinctive business, reduce the similarity of assets, reduce the business of peers, and thus avoid the risk of passive damage when the systemic crisis is contagious. By streamlining operations and fostering innovation, the digitization process can lead to more efficient internal processes that don’t necessarily augment systemic risk through increased interdependencies. Instead, these advancements may enhance the bank’s ability to manage risks more effectively and maintain profitability without contributing to the potential for systemic contagion. To verify the above inference, further data analysis and mechanism discussion are needed, which just points to the specific direction that the following article needs to expand.

**Table 12:** The relationship between bank efficiency and systemic risk at different levels of digital transformation.

Panel A	(1)	(2)	(3)	(4)	(5)	(6)
	lnSL	lnIR	lnbeta	lnSL	lnIR	lnbeta
LCCE	0.032		0.051		0.082	
	(0.026)		(0.077)		(0.084)	
LCPE		-0.034*		0.007		-0.027
		(0.018)		(0.054)		(0.059)
Size	0.012	0.007	0.133**	0.119**	0.145**	0.126**
	(0.018)	(0.016)	(0.053)	(0.049)	(0.057)	(0.053)
Capr	0.009***	0.010***	0.026***	0.027***	0.035***	0.037***
	(0.003)	(0.003)	(0.009)	(0.009)	(0.010)	(0.010)
ROE	-0.000	-0.000	-0.000	-0.000	-0.000	-0.000
	(0.001)	(0.001)	(0.002)	(0.002)	(0.002)	(0.002)
NII	-0.000	-0.000	0.005***	0.005***	0.004***	0.004***
	(0.000)	(0.000)	(0.001)	(0.001)	(0.001)	(0.002)
NPL	-0.006	-0.005	-0.087***	-0.084***	-0.093***	-0.089***
	(0.006)	(0.006)	(0.018)	(0.017)	(0.019)	(0.019)
Constant	-0.596	-0.433	-3.934**	-3.530**	-4.530***	-3.963**
	(0.506)	(0.468)	(1.528)	(1.414)	(1.658)	(1.535)
Bank_FE	Yes	Yes	Yes	Yes	Yes	Yes
Year_FE	Yes	Yes	Yes	Yes	Yes	Yes
Observations	785	785	785	785	785	785
Adjusted R2	0.620	0.621	0.683	0.683	0.667	0.666
Panel B	(1)	(2)	(3)	(4)	(5)	(6)
	lnSL	lnIR	lnbeta	lnSL	lnIR	lnbeta
LCCE	-0.050		-0.102		-0.152	
	(0.056)		(0.112)		(0.106)	
LCPE		0.101***		-0.190***		-0.089
		(0.033)		(0.067)		(0.064)
Size	-0.114*	-0.059	-0.441***	-0.454***	-0.556***	-0.513***
	(0.061)	(0.055)	(0.123)	(0.112)	(0.116)	(0.106)
Capr	-0.002	0.000	0.009	0.009	0.007	0.009
	(0.010)	(0.010)	(0.020)	(0.020)	(0.019)	(0.019)
ROE	0.001	-0.000	0.001	0.004*	0.003	0.004*
	(0.001)	(0.001)	(0.002)	(0.002)	(0.002)	(0.002)
NII	-0.003***	-0.003***	-0.003	-0.003	-0.006***	-0.006***
	(0.001)	(0.001)	(0.002)	(0.002)	(0.002)	(0.002)
NPL	-0.148***	-0.137***	-0.056	-0.089	-0.204***	-0.226***
	(0.031)	(0.031)	(0.062)	(0.062)	(0.058)	(0.059)
Constant	3.172*	1.529	12.535***	13.031***	15.707***	14.560***
	(1.714)	(1.564)	(3.461)	(3.162)	(3.253)	(3.002)
Bank_FE	Yes	Yes	Yes	Yes	Yes	Yes
Year_FE	Yes	Yes	Yes	Yes	Yes	Yes
Observations	416	416	416	416	416	416
Adjusted R2	0.673	0.680	0.701	0.706	0.590	0.589

Notes: This table regresses the bank’s system risk and its components on bank liquidity creation efficiency and bank-specific controls. Panel A represents the results of banks with high digital transformation level. Panel B represents the results of banks with low digital transformation level. Standard errors clustered at bank level. are reported in parentheses where *,** and *** denote statistical significance at 10%, 5% and, 1% levels, respectively.

## 7 Conclusion, implications, limitations, and future research direction

By introducing a measure of bank liquidity creation efficiency and decomposing systemic risk into complementary parts, we explored the relationship between bank liquidity creation efficiency and risk in a deeper perspective. A double-edged effect of bank liquidity creation efficiency for China’s banks is revealed in our work. That is, bank liquidity creation profit efficiency improvement alleviates individual risk while amplifying systemic linkage. We also show that higher bank liquidity creation profit efficiency is associated with more investment in the real estate industry and higher risk-taking level on loans but is not associated with higher managerial quality. Only banks with a higher level of digitization have not experienced an increase in systematic linkage with the financial system while profit-seeking. This indicates that banks’ digital transformation and development may help mitigate the double-edged sword effect of increased profit efficiency.

Although this paper highlights the potential risk behind the improvement of bank liquidity creation efficiency for banks in China, it has global implications. First, China’s economy is tightly connected with the world, and some global systemically important banks are located in China. Our findings may help worldwide regulators understand the latent risk of the banking system. Second, the double-edged effect of bank liquidity creation efficiency on risk may not be typical in China. It could also be a common feature for other emerging markets, where the equity market is not mature enough, and indirect financing like banks is predominant.

Risk prevention is an eternal theme of the financial industry. The paper’s conclusions have significant policy implications for the critical balance between risk and efficiency that banks must navigate during periods of development. These findings reveal the dual-edged nature of bank profit efficiency, highlighting how efficiency enhancement can exacerbate systemic risk through increased interconnectedness. Specific regulation policy can be developed based on the following aspects: First, regulators must pay attention to the double-edged effect of bank efficiency and provide special guidance and supervision of bank real estate loans to avoid severe reliance on this industry. Second, the bank regulation framework needs structure change according to the current driving force of systemic risk. Our findings indicate channels through which bank efficiency chasing contributes to systemic risk—systemic linkage rather than individual tail risk. Regulatory authorities must reassess the risk contagion and resonance effects instigated by bank liquidity creation activities. It is imperative to adjust and enhance the regulatory framework, focusing on the governance of bank profit efficiency maximization operations that present latent risk contagion concerns. Third, regulation authorities can adopt differentiated regulatory approaches for different types of banks and orderly dispose of high systemic linkage businesses and institutions. Small and medium-sized banks are more vulnerable to systemic linkage risk. Fourth, governments must firmly support the implementation of digital transformation strategies. Infrastructure construction is a common strategy to stimulate economic growth in developing countries, especially in their early stages of development. Our finding underscores the role of digital infrastructure as a pivotal factor in the modernization of the banking sector, facilitating a balance between profitability and systemic stability.

However, this study still has limitations. The methodologies employed to measure systemic risk have, thus far, primarily concentrated on listed banks. Acknowledging that the assets managed by listed banks constitute a significant portion of China’s banking industry, their dominance underscores their representativeness concerning the sector’s comprehensive growth. But, overlooking the behavioral characteristics of smaller and medium-sized banks made us fail to offer a more complete depiction of risk within the financial system. Given the above limitations, we argue for further research to expand systemic measurement methodology, using various data sources to illuminate additional facets of systemic risk.

## Supporting information

S1 Table Category and weight of calculating liquidity creation. (PDF)

S1 Data (XLSX)
