# Supplementary material for: The double-edged effect of bank liquidity creation efficiency on systemic risk: Evidence from China
Source: PLoS One. 2024 Nov 14;19(11):e0313208. doi: 10.1371/journal.pone.0313208 (PMC11563395; doi:10.1371/journal.pone.0313208)
Supplement: S1 Table — (PDF) [file pone.0313208.s001.pdf]

# Supporting information

Table 1: **Category and weight of calculating liquidity creation.**

| | | |
|---|---|---|
| Asset | | |
| Illiquid (weight =1/2) | Semiliquid (weight =0) | liquidi (weight =-1/2) |
| Other assets | Net bank loans and loans | Deposit to the Central Bank |
| Equity Investment | Reverse repurchases, borrowed securities, and cash collateral | Derivative financial instruments |
| Residential Mortgage Loan | Consumer Loan | Trading assets measured at fair value |
| Corporate Loan | | Financial assets: available for sale |
| Other loans | | Financial assets: Hold to maturity |
| Bad debt reserve | | Other Securities |
| Deferred tax assets | | Commission receivable |
| Investment real property | | |
| Fixed assets | | |
| Insurance Assets | | |
| Current tax assets | | |
| Stop business | | |
| Foreclosure / owned other property | | |
| Goodwill<br>Other intangible assets | | |
| Equity and Liabilities | | |
| Illiquid (weight =-1/2) | Semiliquid (weight =0) | liquidi (weight =1/2) |
| Long-term borrowing and bonds (greater than 1 year) | Savings deposit | Current deposit |
| Other long-term loans | Time deposit | Other customer deposits |
| Other liabilities | Bank deposit | Derivative financial instruments |
| Stop business operations | Other wholesale deposits | Trading liabilities |
| Insurance liability | Long-term borrowing and bonds (less than 1 year) at historical cost | Commissions payable |
| Current Tax Liabilities | Financial liabilities calculated at fair value | |
| Deferred tax liability | Repurchase Agreement, loan securities, cash mortgage | |
| Reserve fund | | |
| Other Deferred Liability | | |
| Subordinate liabilities | | |
| Total Equity | | |
